# Improved resolution in fiber bundle inline holographic microscopy using multiple illumination sources

**DOI:** 10.1364/BOE.516030

**Published:** 2024-02-09

**Authors:** Michael R. Hughes, Callum McCall

**Affiliations:** Applied Optics Group, School of Physics and Astronomy, University of Kent, Canterbury, Kent, CT2 7NH, United Kingdom

## Abstract

Recent work has shown that high-quality inline holographic microscopy images can be captured through fiber imaging bundles. Speckle patterns arising from modal interference within the bundle cores can be minimized by use of a partially-coherent optical source such as an LED delivered via a multimode fiber. This allows numerical refocusing of holograms from samples at working distances of up to approximately 1 mm from the fiber bundle before the finite coherence begins to degrade the lateral resolution. However, at short working distances the lateral resolution is limited not by coherence, but by sampling effects due to core-to-core spacing in the bundle. In this article we demonstrate that multiple shifted holograms can be combined to improve the resolution by a factor of two. The shifted holograms can be rapidly acquired by sequentially firing LEDs, which are each coupled to their own, mutually offset, illumination fiber. Following a one-time calibration, resolution-enhanced images are created in real-time at an equivalent net frame rate of up to 7.5 Hz. The resolution improvement is demonstrated quantitatively using a resolution target and qualitatively using mounted biological slides. At longer working distances, beyond 0.6 mm, the improvement is reduced as resolution becomes limited by the source spatial and temporal coherence.

## 1. Introduction and background

Inline holographic microscopy is a simple and widely-used technique for capturing high-resolution images of microscopic samples without the need for a microscope objective. Conventionally, the sample is illuminated by a diverging or collimated laser beam. Light scattered by the sample interferes with unscattered portions of the beam, creating an interference pattern which is then directly recorded by a camera. This hologram can be numerically back-propagated to recover the intensity distribution (i.e. an image) at any desired plane with the sample.

The unscattered portion of the beam fulfils the role of the ‘reference beam’ in traditional interferometry. Inline holography therefore works best when the sample is sparse, and is normally used for transmission imaging. Partially coherent sources, such as a spatially filtered light emitting diode (LED), can be used instead of a laser, providing that the distance between the sample plane and the camera is kept short, typically up to a few millimetres [1]. Unlike techniques such as off-axis and phase-shifting holography, inline holography suffers from the twin artefact [2] and cannot directly recover the quantitative phase of the sample without iterative processing or machine learning [3]. However, it has the advantages of being simple, low-cost and robust, with potential applications in fields such as diagnostic imaging [4] and water sample monitoring [5].

Recently, several studies have investigated performing holographic microscopy through fibers. This could have several potential advantages. Firstly, images could be captured in small or confined spaces, turning the microscope into a probe that could, for example, be used endoscopically. Fibers could be plugged into devices such as microfluidic chips, and several fibers could be placed in closer proximity than cameras. Secondly, fibers can be used under-water or at a greater range of temperature than cameras without the need for high-cost protective measures.

One family of approaches involves capturing holograms through the core of a multimode fiber (e.g. [6]). This allows the quantitative phase to be recovered, but via complex methods that require measuring, in some way, the transmission matrix of the fiber, and are generally quite sensitive to fiber bending and other disturbances. An alternative approach is to use fiber imaging bundles, which contain, typically, tens of thousands of fiber cores. These act as image intensity conduits, and have traditionally been employed in endoscopes and more recently in endoscopic microscopes [7]. Since each core supports a few modes at visible wavelengths, and each core will have a slightly different optical path length which varies as the fiber bundle is bent, fiber bundles cannot be used to relay phase information directly. That it to say, the magnitude of a field projected on one end will be faithfully transmitted (up to a pixelation artefact), but the phase information will be distorted. Therefore a fiber bundle cannot simply be placed in one arm of an interferometer. However, there are several approaches for recovering the phase or performing numerical refocusing.

Historically, Coquoz et al. [8] demonstrated inline holographic reflectance mode imaging of a non-biological sample through a fiber bundle, with illumination from a Helium Neon (HeNe) laser routed through a single mode fiber running parallel to the bundle. Later, it was shown that phase contrast and even quantitative phase recovery can be achieved in reflectance mode using multiple oblique illumination fibers [9,10], although it was not clear if numerical refocusing would be possible. Wurster et al. [11] achieved holographic imaging of a human finger through a rigid fiber bundle placed in one arm of a Michelson interferometer, with a tuneable laser used to average over multiple speckle realisations [12]. There is also a large body of work on remote focusing through fiber bundles by compensating for the variations in phase using prior calibrations (see Ref. [13] for a review) but these suffer from the challenge of requiring a new calibration to deal with any bending of the fiber. While recent work has shown some promise that these challenges could be overcome (e.g [14,15]), the technology remains immature and may be too complex for many practical applications.

In 2021, we demonstrated that *transmission mode* inline holographic microscopy is possible in a simple and robust way by capturing inline holograms using a fiber bundle and routing them to an external camera [16]. As with conventional inline holography, this allows numerical refocusing (albeit with the twin image artefact) but not single-shot quantitative phase recovery. Illumination was by an LED coupled to a multimode fiber to minimize speckle noise from modal interference within the fiber bundle cores. Partial coherence limits the numerical refocusing distance to approximately 1 mm from the fiber tip before the resolution begins to degrade significantly [16]. As a transmission mode technique, this is not designed for in vivo medical imaging, although recently Badt et al. [15] demonstrated that placing a partial reflector at the distal end of the fiber allowed for reflectance mode imaging, as well as coherence gating and quantitative phase retrieval via phase shifting. Sun et al. [17] have also subsequently demonstrated phase recovery and numerical refocusing through a fiber bundle using the FAST (far-field amplitude-only speckle transfer) technique, but with the need for a complex numerical calibration (taking several minutes) that would not be robust to fiber bending.

The present article builds on our recently demonstrated approach of capturing inline holograms through a fiber imaging bundle [16]. One of the disadvantages of the system we reported previously is that the resolution is limited, for short working distances, by the finite spacing between the cores in the bundle. This is analogous to the camera pixel size in conventional inline holography, although with some differences in terms of the spatial arrangements (the cores are packed quasi-hexagonally rather than in a rectangular grid) and the lower fill factor (there is cladding between the cores). In traditional inline holography systems using a laser, it is possible to overcome the limit due to the pixel size by using a diverging source and increasing the sample to camera distance, resulting in a magnified hologram with respect to the pixel pitch. With partially-coherent sources, the sample to bundle separation must be kept short, and so the bundle core spacing becomes a limiting factor in the resolution. In the case of fiber bundle inline holography, it is the pitch of the fiber bundle cores that limits the resolution rather than the camera pixel size, as the proximal end of the bundle can be imaged onto the camera with arbitrary magnification. The highest resolution fiber bundles have a core spacing of approximately 3 µm, which we showed previously limits the resolution to approximately 5 µm, somewhat poorer than with camera based systems where cameras with pixel sizes of under 2 µm are readily available.

Several previous publications have suggested that it is possible to improve the resolution of fluorescence (i.e. non-holographic) images acquired through fiber bundles by combining multiple images where the bundle has been shifted with respect to the sample. If the probe is in motion then some improvement may be obtained simply by combining overlapping frames [18,19]. Vyas et al. [20] collected multiple line-scan confocal fluorescence images with the bundle shifted using a piezo-electric actuator and combined them via linear interpolation to improve the resolution by a factor of two. Chang et al. [21] proposed a similar approach using a piezo-tube, although without interpolation or a direct demonstration of the achieved resolution. A similar effect can be achieved by rotating rather than translating the bundle [22].

These methods rely on some movement between the bundle and the sample, which is the only way to shift the bundle pattern with respect to the sample when performing fluorescence imaging. However, as inline holography is a transmission imaging modality using coherent (or partially coherent) light it is possible to shift the hologram without moving either the bundle or the sample. Instead, adjusting the position of the illumination is sufficient. This could be achieved either by physically moving the illumination fiber or by having a number of separate fibers and illuminating through them sequentially. The latter approach was adopted by Bishara et al. [23] for improving the resolution of camera-based inline holographic microscopes using conventional iterative pixel super-resolution techniques.

In this paper we show that a bundle of illumination fibers can be used to sequentially form holograms which are slightly shifted with respect to the fiber bundle. Unlike the camera based work of Bishara et al. [23], which relied on slow iterative reconstruction techniques, the low fill factor of the fiber bundle means that we are able to approximately double the resolution of refocused images by using linear interpolation between the cores of the shifted holograms to produce a higher resolution hologram. Following a one-time calibration, this allows us to produce resolution-enhanced images in real time, and we demonstrate here equivalent frame rates of up to 7.5 Hz.

## 2. Methods

**Fig. 1.**
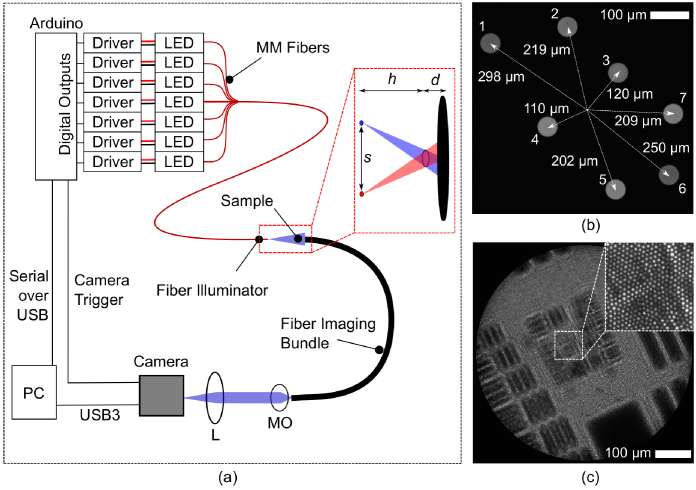
(a) Schematic of system for resolution enhanced inline holographic microscopy through a fiber bundle. L: tube lens, f = 100 mm; MO: microscope objective, 10X. The inset illustrates how the different illumination fibers project holograms to different positions on the fiber bundle. (b) Image of end of illuminator, showing the 7 illumination fibers and their distances from the centre of the group. (c) Typical raw hologram showing pixelation due to fiber bundle cores.

A schematic of the system is shown in Fig. 1(a). Holograms are captured by a 1.5 m long fiber imaging bundle (Fujikura FIGH-30-650S) with approximately 30,000 cores inside an active imaging area with a diameter of 600 µm and an average core-core spacing of approximately 3 µm. At the proximal end this is imaged onto a monochrome CMOS camera (FLIR Flea3 FL3-U3-13S2C-CS) via a 10X infinity corrected microscope objective (Thorlabs RMS10X) and a 100 mm focal length tube lens (Thorlabs AC254-100-A-ML). Magnification between the bundle and the camera is a factor of approximately 6, meaning that the 3.7 µm camera pixels are imaged to a size of 0.62 µm at the plane of the bundle, giving approximately 970 pixels across the diameter of the bundle, and approximately 5 pixels per core-core spacing.

To assemble the multi-fiber illuminator, eight lengths of multimode fiber (Thorlabs FG050UGA, 50 µm core, 125 µm cladding diameter, 0.22 NA) were cut. One end of each fiber was stripped of its 250 µm diameter coating and these were all epoxied inside a single SMA (SubMiniature version A) connector with a small length of epoxied fibers protruding. The end of this bundle of fibers was then polished flat, leaving eight fiber ends within an approximately 550 µm diameter circle. One fiber was damaged during fabrication, leaving seven functional fibers for conducting the study. The other end of each fiber was connectorized with an FC/PC connector.

Seven royal blue LEDs were attached to heat sinks and mounted behind FC/PC adaptors using a custom 3D printed assembly, allowing each of the fibers in the multi-fiber illuminator to be coupled to an LED. Each LED was connected to its own dimmable LED driver, with the dimming control wires each connected to a digital output channel of an Arduino Mega board, allowing each LED to be turned on or off in sequence. The Arduino also generated a trigger signal for the camera via an additional digital output. In normal operation, the Arduino was used to turn each LED on in sequence, with a trigger pulse sent to the camera each time an LED was turned on. At the end of the sequence an additional camera trigger was also sent with all LEDs turned off - this gave an image with very low intensity which was used for synchronisation. Average power delivered by an individual fiber was 37 µW, although variations in coupling resulted in powers ranging from 27 to 43 µW across the seven fibers.

The SMA-connectorized end of the multi-fiber illuminator was mounted 30 mm from the sample, and the distal end of the fiber bundle was then mounted close (typically less than 1 mm) from the sample. Samples were mounted between the fibers on a 3D-printed mount connected to a 3-axis translation stage for positioning. As illustrated in the inset of Fig. 1(a), illuminating with different LEDs results in a shift of the projected hologram with respect to the bundle.

A composite image of the connectorized tip of the illuminator is shown in Fig. 1(b). This image was obtained by imaging the tip of the illuminator onto the camera using the same objective/tube lens arrangement as used for the fiber bundle. Images were acquired with each LED turned on in turn, and then a maximum intensity projection across the seven images was taken to generate a single image showing all of the fibers. The distance of each fiber from the center of the group is indicated. The resulting shift, s′ of the hologram of an object between illumination of any two fibers is given by

(1) $$s\prime =s\frac{d}{d+h},$$

where s is the separation between the fibers, h is the distance from the illumination fiber to the object and d is the distance from the object to the fiber bundle. For results presented below, h was set to approximately 30 mm, while d was in the range of 0.1 to 0.6 mm).

Images were streamed from the camera to a PC via a USB cable. An example of a raw image is shown in Fig. 1(c), where the core pattern can clearly be seen in the zoom inset. All processing times given below were for a PC with an Intel Core i7-7700 processor (4 core), 24 GB RAM, and GeForce RTX 2060 GPU, using code written in Python 3.8. A copy of all code is available in the repository accompanying this paper [24] and the key elements of the code have been integrated into the open source libraries PyFibreBundle [25] and PyHoloscope.

### 2.1. Bundle image pre-processing and core pattern removal

A calibration image is first acquired with no sample in the field-of-view. The location of each fiber core is determined by the following method:

1. Apply a Gaussian smoothing filter with sigma equal to 1/5th the estimated core-core spacing to reduce modal patterns within cores.
2. Apply a morphological dilation with a circular structuring element 3 pixels in diameter.
3. Subtract the dilated image from the original. As dilation does not change the value of local maxima, the centers of each core will then have a value of zero.
4. Threshold the result to discard all but the local maxima.
5. Apply a further morphological dilation with circular structuring element with a diameter equal to 1/3rd of the estimated core spacing. This has the effect of merging maxima which are within a single core which sometimes occur because of residual multimodal effects.
6. Find the centroid of all connected regions - these are the locations of the centers of each core.
7. Remove any detected cores outside the known radius of the bundle.

To reconstruct a de-pixelated hologram from a single raw hologram captured through the bundle, triangular linear interpolation [25] is performed between the intensity values of each core, resulting in a hologram on a rectangular pixel grid. In the one-time calibration stage, which typically takes less than 1 second, a Delaunay triangulation is formed over the core locations. A reconstruction grid size is chosen and, for each pixel in the reconstruction grid, the enclosing triangle of fiber cores is identified. The location of each pixel within its triangle is stored in barycentric co-ordinates. To process subsequent holograms, the intensity of each core is extracted from the raw image by taking the pixel values at the previously calculated core positions. The intensity value for each pixel in the reconstructed hologram is then obtained by triangular linear interpolation between the intensities of the three cores making up the vertices of the triangle enclosing that pixel. Due to the pre-computation in the calibration stages, this only takes approximately 2 ms per image. The intensity of pixel j in the hologram, $p_{j}$ , is given by

(2) $$p_{j}=\sum_{k=1}^{3}c_{k}b_{j,k},$$

where $b_{j,k}$ are the barycentric co-ordinates recorded for reconstruction grid pixel j in the calibration stage and $c_{1}$ , $c_{2}$ and $c_{3}$ are the intensities of the three surrounding cores [25].

Background subtraction is commonly employed in inline holography to remove intensity variations in the illumination beam, creating what is termed as a ‘contrast hologram’. This is essential prior to numerical refocusing to avoid edge artefacts. Flat-fielding is performed to remove inhomogeneities in the illumination and also to correct for variations in transmission between the fiber cores. Both corrections are achieved by recording the value of each core in the calibration image. During reconstruction of a hologram, the corrected intensity for each core, c′ , is given by:

(3) $$c\prime =\frac{b-c}{b}$$

where c is the uncorrected intensity and b is the intensity of the core in the calibration image. Note that this has the effect of inverting the contrast, i.e. areas of absorption have higher pixel values.

### 2.2. Resolution enhancement

To obtain resolution enhancement, a stack of images is acquired with each LED fired in turn. The speed at which this can be done is limited by the exposure time required to obtain good signal level and the frame rate of the camera. With a 3 cm fiber-to-sample distance, a camera exposure of 25 ms was then required to obtain an image which used the full well-depth of the camera with no hardware gain. In triggered mode this limited the camera frame rate to 20 fps and hence a net resolution-enhanced image frame rate of 2.5 fps. A higher frame rate (up to the camera-limited raw frame rate of 60 fps and hence a net frame rate of 7.5 fps) could be obtained by increasing the camera hardware gain, in exchange for a small increase in noise.

Each illumination using a different LED creates a shifted hologram on the fiber bundle face. This shift is dependent on the axial location of the sample (see Section 3.2 for more discussion of this) but is otherwise constant in time. Therefore once an initial calibration is completed the reconstruction of resolution-enhanced images from each set of shifted holograms is fast (approximately 50 ms). The shifts are relatively small because the illuminator to sample distance is much larger than the sample to bundle distance, by at least a factor of 50 for all results presented here, so that the change in angle of rays striking the bundle is very small, less than $1^{\circ }$ . To a very good approximation, the multiple holograms are therefore pure translations of each other, without any additional distortion.

In the first stage of the calibration, each shifted hologram is reconstructed (i.e. core pattern removed) as described as above. The shift between the images is then determined using normalized cross correlation (NCC). To avoid edge effects, the NCC is taken between a central square template one quarter of the bundle diameter and a central square reference image one half the bundle diameter, so that the edges of the bundle are not visible in either. For each shifted image, the recorded core locations are then shifted so that they lie at the true position on the hologram that they sample. This creates a denser set of sampling points over the hologram. A Delauany triangulation is then formed over the full set of corrected core positions from all shifted holograms. As for a single image, the reconstruction grid pixels are then expressed in barycentric co-ordinates with respect to the three enclosing cores, but this time using the full set of shifted cores.

To reconstruct the hologram, core intensities are extracted from all shifted images and triangular linear interpolation is then performed as before, but now over this denser set of points, resulting in a higher resolution hologram. In practice, there are several further considerations to achieving a good quality reconstruction. As discussed below, each LED results in a slightly different output power and distribution of light onto the sample. If this effect is not accounted for then the reconstructed image appears noisy, as there is a variation in pixel intensity depending on which shifted images it was interpolated from. To avoid this problem, a calibration image (i.e. with nothing in the field-of-view) is acquired using each LED. This allows each shifted hologram to have an independent background subtraction and flat-fielding applied, and for each image to be normalised to have the same mean intensity.

The resolution enhancement takes approximately 14 ms and hence is suitable for real-time use at high frame rates. Optimized Python code for performing the enhancement is included in the PyFibreBundle library [25] and a copy has been archived with this article.

### 2.3. Numerical refocusing

Once the de-pixelated hologram (conventional or resolution enhanced) is obtained, this can then be numerically propagated to obtain an image focused at the desired plane within the sample. This was achieved using the angular spectrum method, whereby the hologram is decomposed into a set of plane waves by taking a 2D Fourier transform. The phase accrued by each plane wave in propagating a distance z is then given by [26]:

(4) $$\Delta \phi (u,v)=\frac{2\pi z}{\lambda }\sqrt{1-{\left(\lambda u\right)}^{2}-{\left(\lambda v\right)}^{2}}$$

where λ is the central wavelength, u,v are spatial frequency coordinates, and z is the refocus distance. Numerical refocusing of a hologram H(x,y) to obtain an image I(x,y) at distance z is then achieved via

(5) $$I(x,y)=F^{-1}[F\{H(x,y)\}\text{exp}\{i\Delta \phi (u,v)\}]$$

where F denotes a 2D Fourier transform and $F^{-1}$ its inverse, and x and y are the pixel co-ordinates.

Even following background subtraction, there is often a step in intensity at the edges of the bundle in the reconstructed hologram. This results in ringing artefacts in the refocused image. To minimize this, a circular cosine window was applied to the hologram prior to numerical refocusing, of the form:

$$f(r)=\left\{ \begin{array}{ll} 1, & if\;r<R\\ 0, & if\;r>R+w\\ \text{cos}\;{\left[\frac{\pi }{2w}(r-R)\right]}^{2}, & otherwise \end{array}\right.$$

where r is the distance from the center of the bundle, R is the bundle radius, and w determines the width of the smooth edge, set as 5 pixels.

Windowing and numerical refocusing of an 800×800pixel hologram can be achieved in 40 ms (CPU only) or 18 ms (using the GPU). Optimized Python code for performing these operations is included in the PyHoloscope library and a copy has been archived with this article.

### 2.4. Shift calibration and calibration look-up-table

As explained above, the magnitude of the shift between holograms depends on the distance of the sample/object creating the hologram from the fiber bundle (the ‘depth’ of the object). The relationship between the object depth and the magnitude of the shifts is fixed by the positions of the fibers in the multi-fiber illuminator and its separation from the fiber bundle. Providing these are fixed, the shifts are then only linearly dependent on the depth. Hence, once the shifts have been measured for an object at at least one depth, the correct shifts for an object at another depth can be estimated without measuring them again. This has the benefit that the shifts can be measured once using a single-layer object, such as a resolution target, rather than needing to be determined using every object to be imaged. This concept is experimentally verified in Section 3.2.

When imaging volumes, objects at different depths will exhibit different shifts, and so there is no single calibration which is correct for all depths. In practice, the resolution enhancement should always be calibrated for the intended numerical refocus depth. Objects not at this depth will be blurred in any case and the resolution enhancement will provide no improvement to the refocused image, whether the depth of the calibration is correct or not. As the refocus depth is changed, for example to image an object at a different depth, a different resolution enhancement calibration is then needed. Since each calibration takes several seconds to perform, this process is too slow for applications where there is a need to rapidly change the refocus depth, for example to allow the user to adjust the focus in real-time. However, this drawback can be mitigated by pre-generating a look-up-table (LUT) of calibrations for a range of depths, and then using the closest calibration to the requested refocus depth. The use of such a LUT is demonstrated in Section 3.3.

## 3. Results and discussion

### 3.1. Shifting sample

**Fig. 2.**
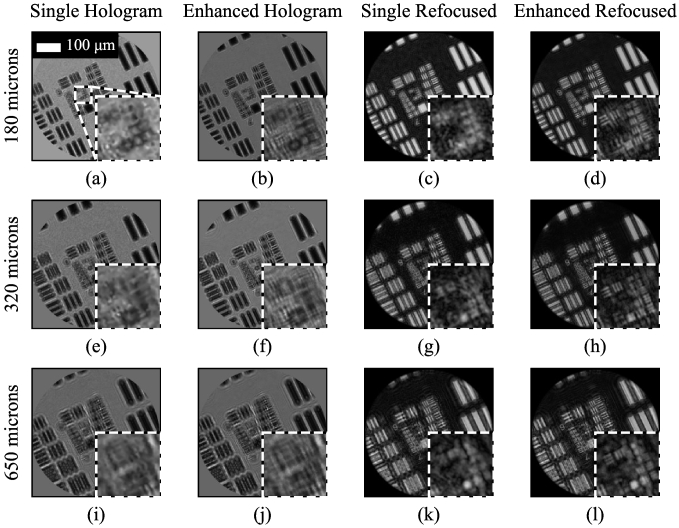
Resolution enhancement by manually shifting resolution target. Holograms and numerically refocused images, with and without resolution enhancement, shown for USAF resolution target at three different distances from the fiber bundle (180, 320 and 650 µm). The scale bar in (a) applies to all images.

To verify the best-case resolution enhancement, irrespective of the specifics of the multi-fiber illuminator, holograms were first acquired using only a single LED from the illuminator, with shifts generated by translating the sample laterally. A United States Air Force (USAF) 1951 resolution target was placed at three distances from the bundle (180, 320 and 650 µm) and holograms were acquired with the object in 16 slightly shifted locations, in approximately a 4×4grid . The mean shift between consecutive images was 6.2 µm and the maximum shift from the center was 17.5 µm; the exact distances are not critical, providing they do not all happen to be integer multiples of the core spacing. The raw holograms were then processed as described above, with the results shown in Fig. 2, where they are compared with reconstruction using only a single image. Both the holograms and the numerically refocused images are shown. Following background subtraction, the raw holograms contain both negative and positive values, while the numerically refocusing involve taking the amplitude, which is why the two sets of images are shown using different gray-level scales.

The improvement in resolution is apparent for the 180 µm distance, with the smallest resolvable group, assessed visually, in the numerically refocused image going from Group 7 Element 4 (G7E4) to G8E4, an improvement from 181 to 362 lines pairs per mm (lp/mm), corresponding an approximately two-fold improvement in the resolution from 5.5 µm to 2.8 µm. This is broadly consistent with previously reported improvements in fiber bundle resolution for bundle-shifting approaches for fluorescence imaging [20]. The improvement for the 320 µm and 650 µm depths is not so great, only to approximately G8E3 and G8E2 respectively. This is because, at larger distances from the bundle, the resolution begins to degrade due to the finite coherence of the LED. Nevertheless, there is still a significant improvement at these depths over using a single hologram.

**Fig. 3.**
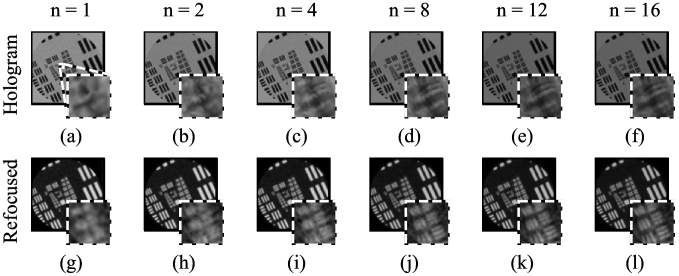
Resolution enhancement by manually shifting resolution target, showing effect of number of images used, from n=1 to n=16 . Top row shows holograms (after core removal and resolution enhancement), bottom row shows refocused images. The insets show a zoom on Group 8 Elements 2-4. Object to bundle distance was 180 µm.

The effect of the number of shifted images for the 180 µm distance is shown in Fig. 3. Much of the improvement is obtained using only 4 shifted images, and there is no visually apparent further improvements beyond using 8 images. While the exact results will depend on the specific shifts, this indicates that the 7 shifts that can be created by the multi-fiber illuminator are sufficient to derive most of the possible improvement.

### 3.2. Resolution target imaging using the multi-fiber illuminator

**Fig. 4.**
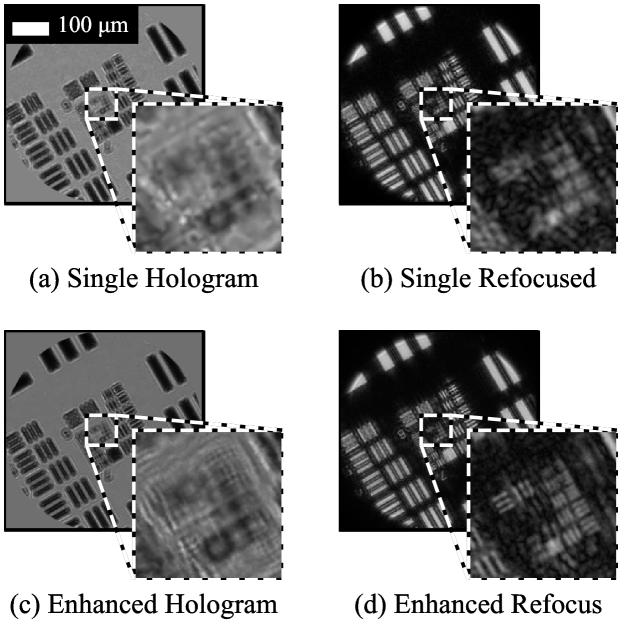
Resolution enhancement for USAF resolution target when using the multi-fiber probe. Enhanced images created using 7 shifted holograms captured in 0.4 s. The USAF resolution target was 240 µm from the fiber bundle. The scale bar in (a) applies to all images.

Results using the same resolution target as above but using the multi-fiber illuminator are shown in Fig. 4. The USAF target was placed 240 µm from the fiber bundle, and a sequence of 7 shifted images were acquired by firing each LED in turn, in addition to a blank reference image (i.e. with all LEDs off). Each shifted hologram had an exposure of 25 ms, and the total acquisition time for all 7 images was 0.4 s, equivalent to a net frame rate of 2.5 fps. As can be seen, the improvement in resolution is approximately the same as for when the target was manually shifted, with G8E3 resolvable in the resolution-enhanced images.

**Fig. 5.**
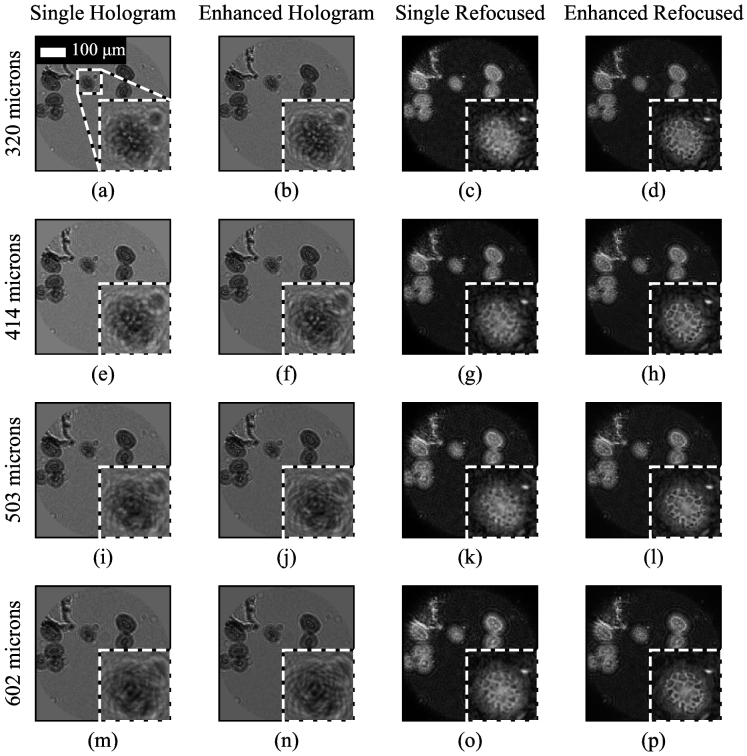
Lillium anther at four different distances from the fiber bundle, with and without resolution enhancement, using the multi-fiber illuminator. Holograms are shown following core removal, background subtraction and flat-fielding. The scale bar in (a) applies to all images. Some of the differences between the images at different working distances are due to the different appearance of the twin image artefact for different sample to bundle distances.

Figure 5 shows an example of resolution-enhanced imaging of lillium anther mounted on a slide behind a glass coverslip with the lillium anther sitting at four different distances from the fiber bundle (305, 414, 503 and 602 µm). The improvement in resolution is not so easy to observe as for the resolution target, but inspection of the zoomed insets shows that features are more clearly resolved and features appear thinner for the resolution-enhanced images

### 3.3. Imaging using predicted shifts and calibration look-up-table

**Fig. 6.**
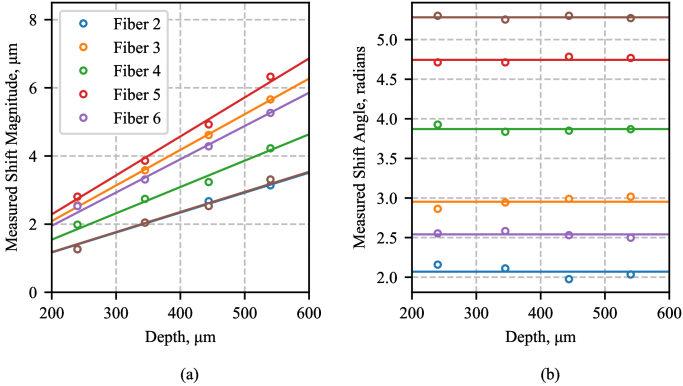
Shifts as a function of depth for six fibers relative to the seventh, measured using the USAF resolution target. (a) Shows the magnitude of the shift and (b) shows the shift direction, expressed as an angle in radians. The linear fits in (a) are forced through the origin, and the fits in (b) are averages across the four depths, since the angle should not change with depth.

As expected, the hologram shift is dependent on the distance of the object from the fiber bundle (the ‘depth’). If there are multiple objects at different depth planes then the contribution to the hologram from each will be shifted by a different amount. This is shown in Fig. 6, which plots the magnitude and direction of the measured shifts at four different depths, obtained using a USAF resolution target. As expected, the shift magnitude is linear with axial position and the direction is constant.

In practice, we always want to use the correct shift for the chosen numerical refocusing distance (i.e. the chosen focus position), regardless of the content of the hologram. Therefore, rather than measuring the shifts for each image, we can perform a one-time measurement using a high-quality imaging target such as the USAF target at one or more depths. (The shift is always known to be 0 for a depth of 0). The target is placed at several depths, with the actual depth found manually, by adjusting the refocus distance until the refocused image is sharpest. For each LED, we measure the vertical and horizontal shift at each of the depths and then perform a linear fit. The expected shift for the hologram due to an object at any depth can then always be calculated.

As discussed in Section 2.4, it is time-consuming to create a calibration for resolution enhancement due to the need to perform a Delaunay triangulation over approximately 200,000 points, typically several seconds. While this is a one-time calibration for an object at a given depth, a new calibration needs to be recreated if there is a significant change in the distance of the sample from the fiber bundle, since this changes the hologram shifts. Creating a look-up-table (LUT) of calibrations for a set of different depths in advance means that, during live-imaging, changes in the depth can be accommodated immediately by using the closest calibration.

**Fig. 7.**
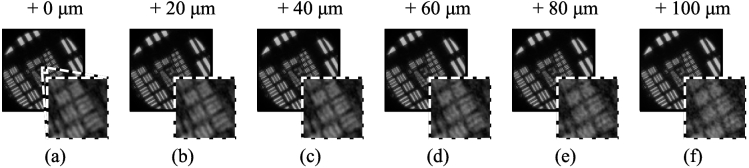
Effect of using a calibration performed at an incorrect depth, in order to determine required density of calibrations. The insets shows a zoom on Group 9, Elements 2-4. As the error increases, from left to right, the resolution enhanced refocused images become increasingly grainy.

To know how many calibrations must be generated in the LUT, the tolerance of the calibration to distance changes needs to be known. To investigate this, the data-set from Fig. 4 was reprocessed using calibrations calculated for increasingly incorrect distances (in increments of 20 µm). Note that numerical refocusing was still performed to the correct distance. As can be seen in Fig. 7, there is no significant degradation with a 20 µm error and little change is visible at 40 µm. To allow a safety margin, LUTs were subsequently created with depth intervals of 20 µm, meaning a maximum error of 10 µm between the true depth and the calibration depth.

**Fig. 8.**
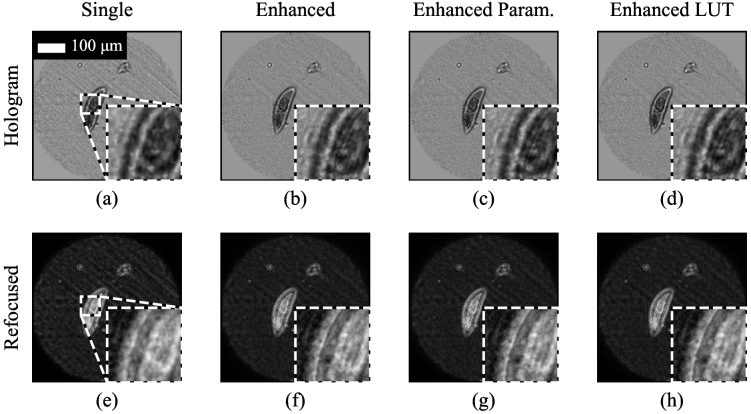
Resolution enhancement of image of paramecium. Top row shows holograms, bottom row shows refocused images. (a) and (e) use a single hologram, (b) and (f) apply resolution enhancement using 7 shifted images, with the calibration performed using the paramecium images. (c) and (g) use parameterized shifts based on images acquired from a USAF resolution target. (d) and (h) use both parameterized shifts and a calibration LUT. The improvement in resolution can be seen in the insets, particularly the thinner outer membrane on the left. The scale bar in (a) applies to all images.

Figure 8 shows an example reconstruction of a paramecium mounted on a slide at a depth of 178 µm. Figure 8(a) shows the reconstruction using a single hologram and panel (b) shows the resolution enhanced image using a calibration performed at the corrected depth using shifts measured with the paramecium images. Panel (c) shows the resolution enhanced image where shifts were estimated from a parameterized depth-to-shift calibration measured using the USAF target, and panel (d) shows the resolution enhanced image using the LUT. In this case, the closest calibration was for a depth of 180 µm and hence, as expected, panels (c) and (d) appear virtually identical. There is also little difference between panels (b) and (c), indicating that here the shift calibration can be performed by either method.

### 3.4. Real-time imaging

**Fig. 9.**
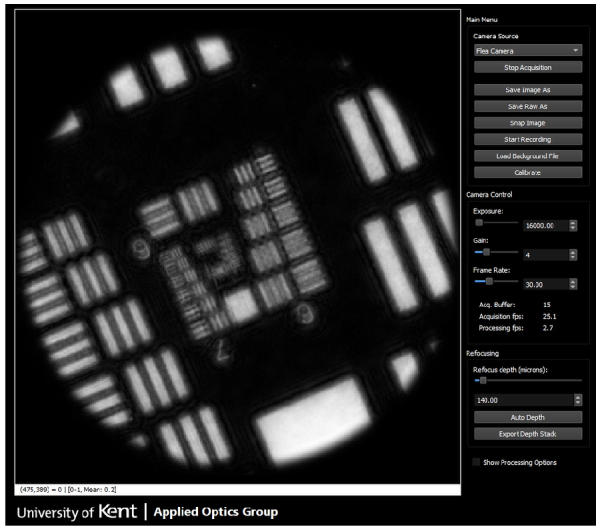
Single frame from Visualization 1 which demonstrates real-time resolution enhancement of a hologram of a USAF resolution target.

Visualization 1 demonstrates that resolution enhancement can be achieved during live imaging. A screenshot is shown in Fig. 9. The video shows a Python-based graphical user-interface which displays processed and refocusing holograms as they are streamed from the camera. A High Resolution USAF imaging target was imaged, initially at a distance of 140 µm from the bundle tip. The video initially shows the refocused hologram without resolution enhancement, at a frame rate of 30 fps. The exposure was set at 16 ms and kept constant throughout.

Resolution enhancement is then turned on, and the resolution visibly improves. With resolution enhancement turned on, raw frames are captured at 25 fps, giving a net enhanced-resolution frame rate of 3.1 fps. In principle a rate of 7.5 fps could be achieved using the camera and the python processing code, but the overheads of the python GUI and the screen recording software resulted in lagging at faster than 3.1 fps. When the sample is translated, motion artefacts are now seen, as expected from a multi-frame approach. Finally, use of a calibration LUT to allow fast refocusing in the GUI is demonstrated. The calibration LUT was built from depths from 100to500µm in steps of 20 µm. The USAF target is shown being moved away from the bundle using a translation stage, and the numerical refocusing then adjusted in the GUI to bring it back into focus, changing which calibration is used from the LUT in the process.

## 4. Discussion and conclusions

The enhancement approach has been shown to improve resolution of fiber bundle inline holographic microscopy by approximately a factor of two. This improvement is obtained only for short working distances; at longer working distances the resolution is limited by the spatial and temporal coherence rather than the fiber bundle core spacing. For the system demonstrated here, using illumination fiber cores at a distance of 3 cm from the sample and a LED bandwidth of approximately 20 nm, a significant resolution improvement is obtained for working distances up to approximately 650 µm. Previous work [16] demonstrated that at a working distance of 1 mm, the intrinsic resolution due to source coherence is poorer than 6 µm, at which point the sampling effect due the fiber bundle becomes negligible and there would be no further benefit to the resolution enhancement technique described here. In principle the working distance could be improved by reducing the illumination core diameter or using a filter to reduce the illumination bandwidth, although this would be at the expense of light throughput and hence would require longer exposures to maintain the same signal-to-noise ratio.

The approach doesn’t require any moving parts at the distal end of the probe because shifted holograms are obtained by illuminating sequentially through different fibers rather than physical movement. The probe can also have a small outer diameter; only the fiber bundle (outer diameter 800 µm) and seven multimode fibers (outer diameter 250 µm, including coating) need to be routed to the distal end.

The work presented here bears some similarity to previous demonstrations of pixel super-resolution applied to camera-based holography, such as Ref. [23]. In this prior work, a bundle of 23 fiber-coupled LEDs was used to generated shifted holograms directly on the camera. Conventional iterative pixel-super-resolution techniques [27] were then used to generate a higher resolution hologram. This did not rely on a low pixel fill-factor, and by using a large number of shifted images [28] it was shown that resolution could be improved from 2 µm to 0.6 µm, a larger factor than demonstrated here. However, conventional pixel super-resolution is an iterative process and generally less suitable for real-time use. With the method reporter here, once a calibration has been performed, resolution enhancement is fast enough for live display of enhanced images. While the calibration does depend on the distance of the object from the bundle, pre-generation of a look-up-table of calibrations across a range of depth allows live refocusing. A potential topic for future work is to investigate whether iterative pixel super-resolution techniques could be used to further improve the resolution in fiber bundle holography.

The images are acquired sequentially and hence the approach is inherently subject to motion artefacts if the sample is in motion. In principle this could be corrected by registering each set of images rather than using a one-time calibration; the sample motion would then provide shifts intrinsically. However, this would require a full calibration to be performed for each image, which would be difficult to perform in real-time.

The probe is not an endoscope or endoscopic microscope is the traditional sense, as it images in transmission rather than reflectance. Potential applications lie in a dippable probe for sampling liquids, or as a clip-on probe for slide imaging. The illumination fibers must be routed to the far side of the sample and then turned round in order to illuminate the volume from the back - in practice this would limit miniaturization of the tip of the probe to approximately double the bending radius of the fibers. Other possible means of reversing the illumination, such as a mirrored surface, could be investigated to enable further miniaturization if required, potentially opening up further applications of the technology.

## Data Availability

Data underlying the results presented in this paper are available in Ref. [24].
